# Fragmented Data, Fragmented Care: Strengthening Health Information Systems for Universal Health Coverage in Somalia

**DOI:** 10.1002/puh2.70416

**Published:** 2026-10-07

**Authors:** Ilyas Abdullahi khalif, Aniso Mohamed Abdi, Yusuf Abdullahi Hubow, Ahmed Mohamed Omar, Nour Ahmed Dahir, Abas Nor Abdi, Sharmake Gaiye Bashir, Nuradin Abdullahi Sheikh Rashid

**Affiliations:** ^1^ Faculty of Health Sciences and Tropical Medicine Somali National University Mogadishu Somalia; ^2^ Center for Health Research and Innovation Somali National University Mogadishu Somalia; ^3^ Faculty of Health Sciences Salaam University Mogadishu Somalia

**Keywords:** digital health, health information systems, health system strengthening, Somalia, universal health coverage

## Abstract

Health information systems (HISs) are essential for achieving universal health coverage (UHC), yet Somalia's health sector remains constrained by fragmented data systems, weak interoperability, limited infrastructure, workforce shortages, and poor governance. This commentary examines the challenges undermining Somalia's digital health transformation and argues that fragmented information systems perpetuate fragmented healthcare delivery. Despite emerging reforms and adoption of digital platforms, such as DHIS2, progress toward UHC remains limited. This commentary draws on a narrative synthesis of published evidence and policy documents to identify priorities for strengthening integrated and resilient HISs in Somalia. Strengthening integrated, resilient, and interoperable HIS is critical for improving health security, equity, outbreak preparedness, and healthcare delivery in Somalia.

## 1 Introduction

Health information systems (HISs) are a foundational pillar of resilient health systems and a prerequisite for achieving universal health coverage (UHC). Functional HISs underpin disease surveillance, continuity of care, outbreak preparedness, health financing, healthcare planning, monitoring and evaluation, and evidence‐based policymaking, enabling equitable and efficient service delivery across populations [1]. Globally, the digital transformation of health systems has emerged as a critical strategy to close equity gaps and accelerate progress toward UHC, particularly in low‐ and middle‐income countries (LMICs) and fragile settings [2]. However, weak and fragmented HISs undermine healthcare delivery, impede public health responses, and perpetuate disparities in access to essential services [3].

Somalia exemplifies these challenges. Decades of conflict, recurrent humanitarian crises, mass displacement, weak infrastructure, donor dependency, and limited institutional capacity have left its health system among the world's most fragile [4]. Despite recent efforts, including adoption of digital platforms such as DHIS2 and strategic frameworks for health system strengthening, Somalia's HIS remains fragmented because of vertical programs, poor interoperability, insufficient governance mechanisms, and underinvestment in digital infrastructure [3]. These persistent weaknesses constrain the country's ability to monitor health trends, allocate resources efficiently, respond to emergencies, and advance toward UHC.

Despite increasing international attention to digital health, most published studies concerning Somalia have focused on describing individual HISs, digital health implementation, disease surveillance platforms, or broader health system reforms. Few have critically examined how fragmentation across HISs influences continuity of care, health system governance, evidence‐based decision‐making, and progress toward UHC within Somalia. This commentary addresses that gap by integrating evidence from the HISs, digital health, and health systems literature to provide a policy‐oriented synthesis specifically focused on the intersection between fragmented HIS and UHC in Somalia. In doing so, it offers a health systems perspective and identifies context‐specific priorities for strengthening governance, interoperability, digital infrastructure, workforce capacity, and data stewardship to support resilient health system development.

Although selected evidence is drawn from Somaliland and comparable fragile settings where national Somalia‐level data remain limited, the primary focus of this commentary is the broader Somali health system and its implications for achieving UHC.

Therefore, this commentary argues that fragmented and underdeveloped HISs are major barriers to achieving UHC and health security in Somalia. Strengthening interoperable, resilient, and integrated HIS must become a national policy priority to ensure equitable access to quality care for all Somalis [3].

### 1.1 Approach to Evidence Identification

This commentary was informed by a narrative synthesis of published literature and policy documents examining HISs, digital health, and UHC in Somalia and comparable fragile and conflict‐affected settings. Targeted literature searches were conducted in PubMed, Scopus, and Google Scholar, together with reports published by the World Health Organization, Somalia's Ministry of Health, the World Bank, and other international development partners.

Search terms included combinations of “Somalia,” “Somaliland,” “Health Information Systems,” “digital health,” “DHIS2,” “electronic health records,” “Universal Health Coverage,” “health system strengthening,” “health governance,” “data quality,” “interoperability,” and “fragile states.” English‐language publications published between 2015 and 2026 were prioritized, whereas highly influential earlier publications were included where they provided important conceptual or policy foundations.

Sources were screened for relevance to Somalia or comparable fragile settings and selected on the basis of methodological credibility, policy relevance, and their contribution to understanding governance, digital health implementation, HIS performance, and progress toward UHC. Findings from eligible publications were reviewed iteratively and synthesized narratively using thematic analysis. Recurring themes—including governance, infrastructure, interoperability, workforce capacity, data quality, digital transformation, and policy implementation—were identified, compared across sources, and integrated into a health systems perspective to generate policy recommendations relevant to Somalia.

As this article is a commentary, no formal systematic review methodology, risk‐of‐bias assessment, or primary data collection was undertaken.

## 2 Somalia's Emerging Digital Health and UHC Landscape

Somalia has renewed its commitment to UHC through primary healthcare‐oriented reforms, including revision of the essential package of health services (EPHS) and the health pillar of the National Transformation Plan 2025–2029 [5]. The revised EPHS, developed through an inclusive national coordination mechanism, prioritizes high‐impact, cost‐effective services and progressive expansion of coverage, while recognizing severe fiscal, workforce, and governance constraints [6]. International partners, humanitarian agencies, and a large private sector remain central to service delivery, reflecting both support and persistent fragmentation [6].

Somalia's HIS increasingly relies on DHIS2 for routine reporting. Although implementation progress has expanded opportunities for routine health reporting and performance monitoring, persistent challenges remain regarding reporting completeness, data quality assurance, interoperability across providers, and integration of information generated outside nationally coordinated platforms. Nevertheless, paper‐based tools remain widespread, particularly outside better resourced facilities, mirroring patterns observed across fragile African settings [7]. Weak governance and donor‐aligned architectures contribute to parallel, poorly interoperable data that flow between public, private, NGO, and vertical program platforms, undermining national stewardship and data use for UHC [5, 7].

Although progress remains uneven, Somalia has demonstrated gradual expansion of digital health infrastructure and routine health reporting mechanisms in recent years. National reforms supporting DHIS2 implementation and primary healthcare strengthening have improved opportunities for data collection and health system monitoring; however, reporting completeness, interoperability across providers, and integration of private sector data remain persistent challenges. At the same time, increasing mobile connectivity and digital adoption present opportunities to strengthen electronic reporting, support continuity of care, and improve evidence‐informed decision‐making for UHC. Continued investment in measurable indicators of HIS performance and data quality will be necessary to track progress and guide implementation.

At the same time, rising mobile phone penetration, broader connectivity, and early telemedicine and digital health pilots signal real opportunities to integrate electronic medical records, community data, and decision support tools into a more coherent digital public infrastructure for UHC [5]. Realizing this potential requires a decisive shift from projectized, donor‐driven systems toward nationally governed, interoperable digital architectures that align information investments with Somalia's broader UHC and primary care reforms [7].

## 3 Major HIS Challenges in Somalia: A Health Systems Perspective

To strengthen analytical depth, the challenges discussed in this section are interpreted through a health systems perspective that considers governance, infrastructure, workforce capacity, data quality, interoperability, and stewardship as interconnected determinants of health system performance and progress toward UHC. This approach enables examination not only of technological constraints but also of broader institutional and implementation factors shaping HIS effectiveness in Somalia.

### 3.1 Fragmented and Parallel Data Systems

Available evidence from Somaliland suggests that HISs remain characterized by fragmented sub‐systems, heavy dependence on external funding, and limited policy and governance structures, resulting in poor coordination and underutilization of health information for decision‐making [3]. Similar donor‐aligned, project‐based architectures in African countries produce parallel reporting channels, duplication, and incomplete national pictures, undermining planning for UHC [8].

### 3.2 Weak Infrastructure and Limited Digital Connectivity

Evidence from Somaliland and comparable fragile settings indicates that inadequate ICT infrastructure, including unreliable electricity and limited network coverage, remains a major barrier to digital health implementation and effective HIS performance [3]. These weaknesses particularly affect rural and conflict‐affected facilities, limiting use of electronic registries and real‐time surveillance needed for equitable UHC [9].

### 3.3 Human Resource and Technical Capacity Gaps

Evidence from Somaliland suggests that HIS performance is constrained by shortages of adequately trained personnel and limited capacity to analyze and utilize health data effectively [3]. Across African and fragile settings, scarce skilled labor and limited computer literacy similarly restrict sustainable digital health integration [9].

### 3.4 Poor Data Quality, Timeliness, and Reliability

Available findings from Somaliland indicate persistent challenges related to data completeness, timeliness, and reliability, whereas incomplete reporting from private providers and weak validation mechanisms continue to undermine effective use of HISs [3]. Fragmentation across multiple systems drives missing data and undermines surveillance, resource allocation, and outbreak response [10].

### 3.5 Lack of Interoperability and Continuity of Care

Multiple nonintegrated systems, absence of universal patient identifiers, and lack of standards constrain interoperability in LMICs, blocking patient tracking and coordinated referrals [10].

### 3.6 Cybersecurity and Data Governance Vulnerabilities

Somaliland lacks HIS policy and legal frameworks, mirroring broader LMIC gaps in data governance, privacy, and cybersecurity, which threaten confidentiality and public trust in digital tools essential for UHC [3, 7].

Across these dimensions, available evidence suggests that fragmentation in HISs continues to undermine continuity of care and progress toward UHC across the Somali health sector.

## 4 Why Strengthening HIS Matters for Somalia's UHC Agenda

Resilient and integrated HISs are foundational to Somalia's health system transformation and the pursuit of UHC. Robust HISs enable continuity and quality of care by ensuring that patient data, service delivery records, and health outcomes are accurately tracked and shared across providers, reducing fragmentation and improving clinical decision‐making [4]. Enhanced HISs support disease surveillance and outbreak preparedness, as demonstrated by improvements in digital platforms, like DHIS2 and EWARN, which have increased the timeliness of reporting and responsiveness to epidemics [4]. Reliable health data are essential for monitoring maternal and child health, immunization coverage, and resource allocation critical for targeting interventions to vulnerable populations [3]. Strong HISs support evidence‐based policymaking, healthcare financing reforms, emergency preparedness, humanitarian coordination, and accountability across the health sector [4]. Nevertheless, the available evidence base for Somalia remains limited and uneven across regions, highlighting the need for more nationally representative and implementation‐focused studies to evaluate the effectiveness and sustainability of digital health investments. In fragile settings, like Somalia, where health inequities are pronounced and resources limited, integrated HISs are vital for measuring performance, identifying gaps, and ensuring efficient delivery of essential services.

However, strengthening HIS alone will not automatically translate into improved health outcomes unless governance, financing, workforce readiness, and accountability mechanisms are strengthened simultaneously. Experience from fragile and low‐resource settings suggests that digital health investments frequently underperform when implemented as stand‐alone technological interventions without sustained institutional ownership, integration into routine service delivery, and long‐term financing strategies. Therefore, HIS strengthening should be viewed as a system‐level reform rather than solely a technological intervention.

Ultimately, strengthening HIS is not merely a technological upgrade but a strategic investment in resilience, equity, health security, epidemic preparedness, and progress toward the Sustainable Development Goals [1, 4].

## 5 Policy Priorities and Strategic Recommendations

Given Somalia's fragile context, resource constraints, and dependence on external support, implementation of HIS reforms should follow a phased and context‐sensitive approach. Short‐term priorities (1–2 years) should focus on strengthening governance, establishing core data standards, improving routine reporting systems, and reinforcing data quality mechanisms. Medium‐term priorities (3–5 years) should emphasize workforce development, interoperability, and progressive expansion of digital infrastructure. Long‐term priorities (beyond 5 years) should support integrated electronic health systems, sustainable financing mechanisms, and nationwide digital health scale‐up aligned with UHC goals.

### 5.1 Develop an Integrated National Digital Health Strategy

Somalia should adopt a nationally owned digital health strategy that clarifies governance roles across federal and state levels, sets interoperability standards, and aligns donor, humanitarian, and private sector investments [9]. A high‐level digital health steering committee, embedded in the Ministry of Health, should coordinate partners, harmonize platforms, and ensure alignment with UHC and health security priorities [11].

### 5.2 Invest in Digital Infrastructure and Connectivity

Targeted investments in reliable electricity, broadband connectivity, secure cloud or data centers, and basic digital equipment are essential, with priority to primary care facilities in rural and conflict‐affected areas [9]. Leveraging public–private partnerships with telecom providers can reduce costs and expand coverage [11].

### 5.3 Strengthen Health Workforce Capacity

A phased capacity building plan should embed digital health competencies into pre‐service curricula, continuous professional development, and on‐the‐job mentoring [11]. Dedicated HIS cadres at district and facility levels, backed by remote technical support, can sustain routine data use [12].

### 5.4 Improve Data Quality and Governance

Somalia should adopt a core national indicator set, routine data quality reviews, and mandatory reporting standards across all providers [12]. Clear accountability mechanisms, including feedback loops and performance dashboards, can institutionalize data use for planning and resource allocation [12].

### 5.5 Promote Interoperability and Patient‐Centered Care

Progressive implementation of electronic medical records, starting with high‐volume facilities, should be guided by national interoperability standards and a phased national patient identifier [11]. Strengthened digital referral and feedback systems can support continuity of care for mobile and displaced populations [9].

### 5.6 Strengthen Cybersecurity and Data Protection Frameworks

A national digital health legal framework should define data ownership, consent, and privacy protections, supported by minimum cybersecurity standards, encryption, role‐based access, and routine backups [11]. Partner‐supported risk assessments and contingency plans are critical to safeguarding sensitive health data in a fragile, high‐risk environment [11, 13].

### 5.7 Strengths and Limitations

This commentary synthesizes available evidence to highlight the importance of strengthening HISs for advancing UHC in Somalia. However, this commentary is based on secondary literature and policy documents and therefore does not provide primary empirical evidence or systematic review findings. In addition, the availability of Somalia‐specific evidence remains limited in some areas, and several findings draw on experiences from comparable fragile settings, which may limit generalizability across all regions of Somalia.

## 6 Conclusion

Somalia's experience illustrates that fragmented and underdeveloped HISs are not peripheral technical flaws, but core constraints on effective service delivery, health security, and progress toward UHC. Without reliable, integrated, and timely data, efforts to strengthen primary care, target scarce resources, and protect vulnerable populations will remain undermined. Addressing these gaps requires more than isolated digital initiatives; it requires sustained political leadership, clear governance arrangements, predictable financing, and long‐term investment in both the health and digital workforce, underpinned by coordinated action across government, humanitarian, and private actors. Looking forward, building resilient, interoperable, and equitable digital health systems must be recognized as a strategic national priority. Future research should move beyond commentary‐based evidence and prioritize empirical assessment of DHIS2 implementation, interoperability across health information platforms, integration of private sector reporting, digital workforce readiness, cybersecurity preparedness, and the cost‐effectiveness of HIS investments in Somalia. Generating context‐specific evidence in these areas will be essential for informing policy decisions and evaluating progress toward UHC. If pursued with consistency and inclusivity, HIS strengthening can enable Somalia to advance UHC, bolster epidemic preparedness, reduce geographic and social inequities in care, and construct a health system capable of withstanding future shocks and safeguarding population health.

To our knowledge, this commentary is among the first to explicitly examine the relationship between fragmented HISs and progress toward UHC within Somalia. Unlike previous publications that primarily describe digital health initiatives or individual HIS components, this commentary integrates evidence across governance, interoperability, infrastructure, workforce, data quality, and health system performance to demonstrate how fragmentation affects UHC implementation. By synthesizing available evidence through a health systems perspective, the manuscript provides a context‐specific policy framework that can inform future digital health reforms and research priorities in Somalia and other fragile settings.

## Author Contributions

Ilyas Abdullahi khalif conceptualized this topic. Nuradin Abdullahi Sheikh Rashid supervised this project. Yusuf Abdullahi Hubow and Nour Ahmed Dahir conducted literature review. Ahmed Mohamed Omar, Aniso Mohamed Abdi, and Abas Nor Abdi wrote the first draft. Sharmake Gaiye Bashir revised and edited this manuscript. All authors approved the final version of the manuscript.

## Funding

The authors have nothing to report.

## Ethics Statement

The authors have nothing to report.

## Conflicts of Interest

The authors declare no conflicts of interest.

## Data Availability

Data sharing is not applicable to this article as no datasets were generated or analyzed during the current study.
